# Long-term postoperative quality of life in childhood survivors with cerebellar mutism syndrome

**DOI:** 10.3389/fpsyg.2023.1130331

**Published:** 2023-02-24

**Authors:** Kaiyi Zhu, Wei Yang, Zesheng Ying, Yingjie Cai, XiaoJiao Peng, Nijia Zhang, Hailang Sun, Yuanqi Ji, Ming Ge

**Affiliations:** ^1^Department of Cardiology, Shanxi Bethune Hospital, Shanxi Academy of Medical Sciences, Tongji Shanxi Hospital, Third Hospital of Shanxi Medical University, Taiyuan, China; ^2^Division of Cardiology, Department of Internal Medicine, Tongji Hospital, Tongji Medical College, Huazhong University of Science and Technology, Wuhan, China; ^3^Department of Neurosurgery, Beijing Children’s Hospital, Capital Medical University, National Center for Children’s Health, Beijing, China

**Keywords:** cerebellar mutism syndrome, quality of life, PedsQL, risk factors, long-term

## Abstract

**Background:**

To investigate the long-term quality of life (QoL) of children with cerebellar mutism syndrome (CMS) and explore the risk factors for a low QoL.

**Procedure:**

This cross-sectional study investigated children who underwent posterior fossa surgery using an online Pediatric Quality of Life Inventory questionnaire. CMS and non-CMS patients were included to identify QoL predictors.

**Results:**

Sixty-nine patients were included (male, 62.3%), 22 of whom had CMS. The mean follow-up time was 45.2 months. Children with CMS had a significantly lower mean QoL score (65.3 vs. 83.7, *p* < 0.001) and subdomain mean scores (physical; 57.8 vs. 85.3, *p* < 0.001; social: 69.5 vs. 85.1, *p* = 0.001; academic: *p* = 0.001) than those without CMS, except for the emotional domain (78.0 vs. 83.7, *p* = 0.062). Multivariable analysis revealed that CMS (coefficient = −14.748.61, *p* = 0.043), chemotherapy (coefficient = −7.629.82, *p* = 0.013), ventriculoperitoneal (VP) shunt placement (coefficient = −10.14, *p* = 0.024), and older age at surgery (coefficient = −1.1830, *p* = 0.007) were independent predictors of low total QoL scores. Physical scores were independently associated with CMS (coefficient = −27.4815.31, *p* = 0.005), VP shunt placement (coefficient = −12.86, *p* = 0.025), and radiotherapy (coefficient = −13.62, *p* = 0.007). Emotional score was negatively associated with age at surgery (coefficient = −1.92, *p* = 0.0337) and chemotherapy (coefficient = −9.11, *p* = 0.003). Social scores were negatively associated with male sex (coefficient = −13.68, *p* = 0.001) and VP shunt placement (coefficient = −1.36, *p* = 0.005), whereas academic scores were negatively correlated with chemotherapy (coefficient = −17.45, *p* < 0.001) and age at surgery (coefficient = −1.92, *p* = 0.002). Extent of resection (coefficient = 13.16, *p* = 0.021) was a good predictor of higher academic scores.

**Conclusion:**

CMS results in long-term neurological and neuropsychological deficits, negatively affecting QoL, and warranting early rehabilitation.

## Introduction

Cerebellar mutism syndrome (CMS) is a multifaceted complication after posterior fossa surgery in children. Up to 25% of children with posterior fossa tumors are affected by this clinical condition (Robertson et al., 2006). Transient loss of speech is thought to be the primary symptom of CMS and has been of great concern in previous studies. To date, the mechanism of CMS has been elusive, and most studies have concentrated on revealing its risk factors. Medulloblastoma, midline location, younger age, and male sex were thought to be risk factors of CMS (Gronbaek et al., 2021; Yang et al., 2022a). A consensus on CMS was reached in 2016 that included other symptoms in addition to speech deficits, including emotional liability and cognitive and motor dysfunctions, with these persisting longer (Gudrunardottir et al., 2016). However, few studies have reported the long-term condition of children with CMS. And previous studies indicated that the long-term performance of children remains uncertain (Camara et al., 2020; Grieco et al., 2020; Khan et al., 2021).

With the improved survival rate of children with brain tumors, long-term quality of life (QoL) has become a key concern in clinical practice. Posterior fossa tumors are the most common brain tumors in children (Ostrom et al., 2021), and CMS is the most common complication (Ashida et al., 2021). The impact of CMS on children’s QoL remains unknown. The Pediatric Quality of Life Inventory (PedsQL) is designed to measure the core dimensions of health, which include physical, emotional, and social functions, as well as school performance, well covering the domains related to CMS. The inventory includes self-report and parent-proxy report versions, both of which are highly reliable. Several studies on the QoL of children with brain tumors have been conducted, where PedsQL 4.0 were widely used (Eaton et al., 2020; Li et al., 2021; Peragallo et al., 2021). However, similar studies on CMS are scarce.

Similar with CMS, cerebellar cognitive affective syndrome (CCAS) is another complication after posterior fossa surgery, which stresses executive deficit, visuospatial cognition deficit, speech deficit, and emotional deficit. Thus far, the relationship between CMS and CCAS remains controversial (Argyropoulos et al., 2020). Some researchers regard CMS as one aspect of the larger neuropsychological entity of CCAS; however, this perspective was not widely recognized (Sadeh and Cohen, 2001; Schmahmann, 2020). At present, the Posterior Fossa Society^1^ still considers CMS and CCAS as two different but related entities; however, the two diseases share similar deficits in cognitive and emotional domains (Schmahmann, 2020).

The emotional impairment can manifest with social–emotional aberrant behaviors, social cognition impairments, and poor self-monitoring of negative emotion (Clausi et al., 2015; Hoche et al., 2016). The initial papers indicate that affective impairments are transient (Argyropoulos et al., 2020), whereas subsequent work has shown that affective changes can be chronic, especially in adult survivors of childhood cerebellar insults (Steinlin et al., 2003). As Schmahmann has mentioned, the affective component is assessed in a less systematized fashion, with many previous studies employing clinical psychiatric assessment, which is based on DSM diagnosis (Argyropoulos et al., 2020). Although a working group in posterior society have been built to develop an easy-to-use scale to quantify the emotional impairments of CMS, no standardized scale has been published so far. Herein, we expect to assess the affective status with emotional domain of PedsQL, and this may provide useful information for future studies.

The cerebellum has been gradually considered to play a role in cognitive and executive function. Studies of CCAS also revealed that the posterior lobe of the cerebellum was associated with cognitive impairment (Stoodley et al., 2016). In addition, radiation to the posterior fossa is expected to impair cognitive function (Mulhern et al., 2005; Conklin et al., 2008; von Hoff et al., 2008; Aarsen et al., 2009; Moxon-Emre et al., 2014). Therefore, the long-term cognitive performance in children with CMS needs to be investigated further.

The school domain of PedsQL assesses behaviors associated with attention, memory, and learning capability. Previous studies have demonstrated that neurocognitive performance is correlated with QoL outcomes (Kuhlthau et al., 2012). Low school scores may reflect impairments in cognitive functioning, processing speed, and attention, at least in part. Considering its accessibility and simplicity, the school domain was used to reflect the cognitive performance in this study.

Above all, in this cross-sectional study, we aimed to investigate the long-term QoL of children with or without CMS and explore the associated factors that have an impact on QoL.

## Methods

### Inclusion and exclusion criteria

**Figure 1:**
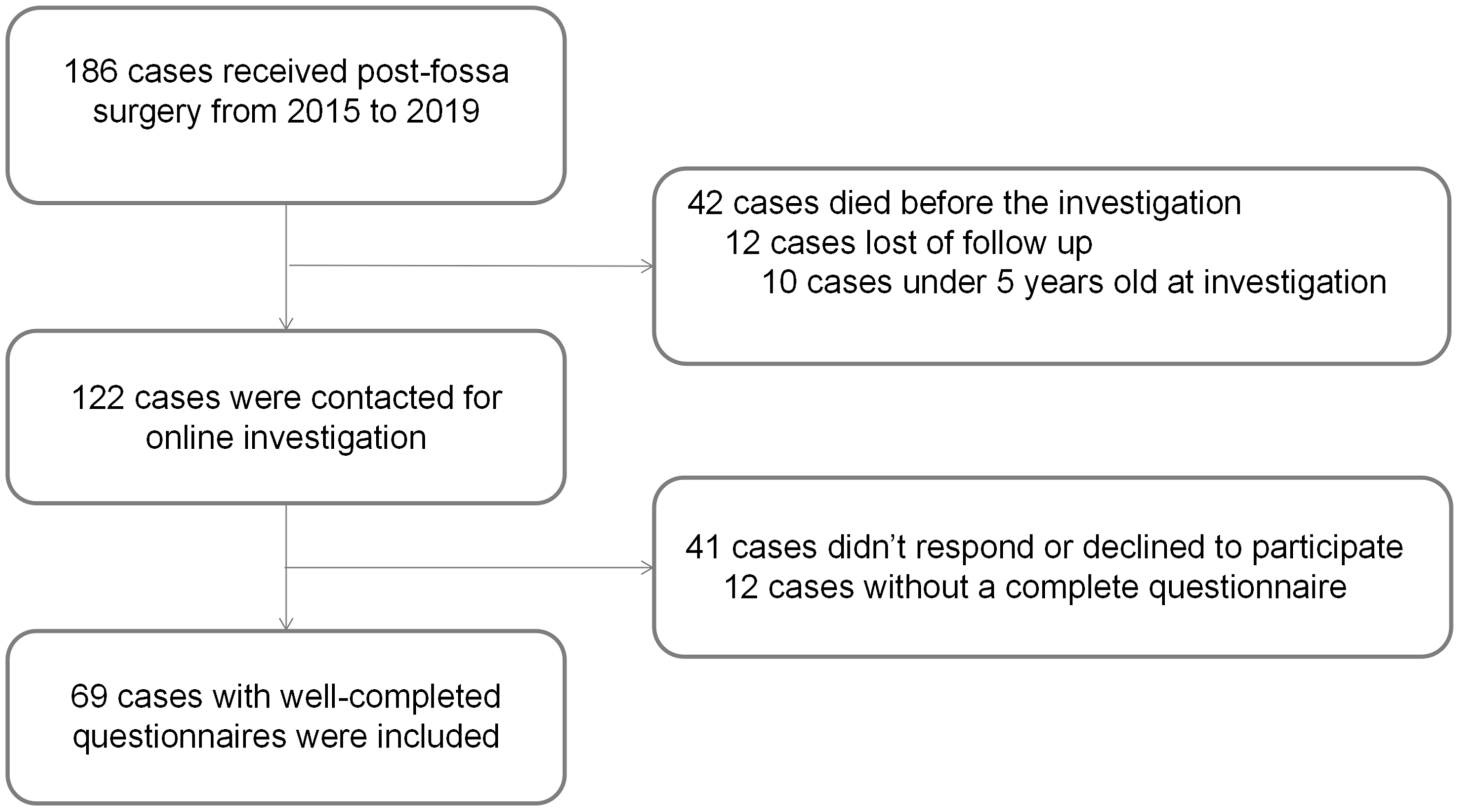
Workflow of the patient selection.

This retrospective study was approved by the Ethics Committee of the Beijing Children’s Hospital (grant number: [2022]-E-076-R). Patients diagnosed with posterior fossa tumors and treated by tumor removal surgery before December 2019 at our hospital were reviewed. The inclusion criteria were (1) diagnosis of posterior fossa tumors by MR and pathology; (2) tumor removal surgery; (3) more than 2 years since surgery date; (4) age 5–18 years at investigation; and (5) consent to participate in this study. The exclusion criteria were as follows: (1) patients who underwent biopsy rather than craniotomy; (2) no survival at follow-up or loss of follow-up; (3) incomplete medical records that influenced the statistical analysis; and (4) CMS status that could not be determined through medical records or phone inquiries. Participants were divided into CMS and non-CMS groups based on their diagnosis of CMS. Figure 1 shows the workflow of patient selection.

### CMS definition

Based on the consensus from the Delphi conference, CMS was defined as mutism or notably reduced speech after posterior fossa tumor removal. Hypotonia, behavior change, and dysphagia after surgery during hospital days were also acquired from medical records or telephone interviews to further depict the details of CMS manifestations. Due to the difficulties of assessing ataxia when patients are in a hypotonia status, we did not include this feature. Behavior change was defined as when the patient presented with irritability or apathy. The onset of mutism was defined as days after the surgery date, and the surgery date was set as day 0. The restoration of speech was defined as being able to speak at least one Chinese word, i.e., “Baba” (dad) or “Mama” (mum), rather than a conversational speech. The duration of mutism was the duration between mutism onset and speech restoration. The aforementioned were collected from medical records and a follow-up database.

### QoL assessment

Patients eligible for inclusion were contacted *via* phone or email. Patients who were willing to participate in this study were sent an online version of the PedsQL 4.0 Generic Core Scale (in Chinese). The PedsQL 4.0, assesses four domains of QoL, including physical, social, emotional, and academic functions. The scale consists of self-report and parent–proxy reports. We chose the PedsQL 4.0, Generic Core Scales because of its frequent use in pediatric literature and its specific brain tumor module. It has been demonstrated that self-reported scores correlate well with proxy-reported scores (Varni et al., 2007; Cheng et al., 2015). Here, we used proxy reports to assess the QoL. Items are reverse scored and linearly transformed to a 0–100 scale. The row score was transformed to the 0–100 range according to the guidance of PedsQL manuals. Higher scores mean better function. The items were listed as an appendix in Supplementary Table S1.

### Clinical variables

Sex, age, tumor size, and ventroperitoneal (VP) placement were obtained from medical records or follow-up information. Ages at surgery and investigation were included in the analysis. Hydrocephalus was defined as Evans index >0.3. The tumor size was defined as the maximum diameter of the three planes. Tumor locations were categorized into midline and lateral. Pathology grades were classified as low grade (World Health Organization [WHO] I–II) and high grade (WHO III–IV). Chemotherapy and radiotherapy treatment information was acquired during the follow-up.

### Statistics

Data analysis was conducted using SPSS (IBM SPSS Statistics for Windows, Version 22.0. Armonk, NY: IBM Corp.). Descriptive statistics for continuous variables and percentages for categorical variables were calculated. PedsQL total scores and sub-scores are reported as the mean and standard deviation. A two-sample *t*-test or Chi-squared test was used to test for group differences. The Pearson’s correlation coefficient was calculated for continuous variables. A forward stepwise linear regression was performed in the multivariable analysis, and variables in the univariate analysis were included. Forward stepwise regression is a stepwise regression approach that starts from the null model and adds variables that improves the model the most, one at a time, until the stopping criterion is met.

## Results

### Patient characteristics

A total of 69 patients who received posterior fossa tumor resection between 2015 and 2019 were included in this study. Of these, 22 patients were diagnosed with CMS after surgery. All patients with CMS restored their speaking abilities (be able to speak conversational speech) prior to the investigation. The median duration of mutism was 6.29 (range: 1–19.43) weeks (see Table 1). Of the patients with CMS, 28.6% restored their speech within 1 month and 76.2% did so within 3 months.

**Table 1:** CMS patient’s characteristics.

CMS group (*N* = 22)	Statistic description
Male (*N*, %)	20, 90.9%
Age at surgery, mean (SD)	6.2 (3.2)
Mutism (*N*, %)	21, 95.5%
Onset of mutism (median, day)	2.5
Duration of mutism (median, week)	6.29
Reduced speech (*N*, %)	1, 4.5%
Hypotonia (*N*, %)	17, 77.3%
Dysphagia (*N*, %)	14, 63.6%
Behavior change (*N*, %)	19, 86.4%

CMS, cerebellar mutism syndrome.

Demographic information and clinical characteristics of the two groups of children are presented in Table 2. The median age at surgery of the cohort was 5.4 [3.0, 8.2] years, and no significant difference was detected between the CMS (5.8 [4.2, 7.7]) and non-CMS groups (5.3 [2.9, 8.6]). Compared with the non-CMS group, the CMS group had a greater proportion of male participants (90.9% vs. 48.9, *p* = 0.002). The two groups showed significant differences in hydrocephalus, tumor location, presurgical VP shunt, extent of resection, surgical routes, pathology, hypotonia, oropharyngeal dysfunction, and behavioral change (*p* < 0.05). Tumor size was evenly distributed between the two groups.

**Table 2:** Patients clinical characteristics.

		Overall (*N* = 69)	non-CMS (*N* = 47)	CMS (*N* = 22)	*p*-Value
Sex, *n* (%)	Female	26 (37.7)	24 (51.1)	2 (9.1)	0.002
	Male	43 (62.3)	23 (48.9)	20 (90.9)	
Age at surgery, mean (SD)		6.0 (3.4)	5.8 (3.4)	6.2 (3.2)	0.649
Tumors size, mean (SD)		48.4 (12.7)	46.2 (14.4)	53.2 (6.0)	0.006
Hydrocephalus, *n* (%)	No	28 (40.6)	25 (53.2)	3 (13.6)	0.004
	Yes	41 (59.4)	22 (46.8)	19 (86.4)	
Midline location, *n* (%)	No	14 (20.3)	14 (29.8)	0 (0)	0.003
	Yes	55 (79.7)	33 (70.2)	22 (100.0)	
VP shunt, *n* (%)	No	56 (81.2)	34 (72.3)	22 (100.0)	0.006
	Yes	13 (18.8)	13 (27.7)		
EOR, *n* (%)	No	10 (14.5)	3 (6.4)	7 (31.8)	0.009
	Yes	59 (85.5)	44 (93.6)	15 (68.2)	
Pathology, *n* (%)	EP	9 (13.0)	3 (6.4)	6 (27.3)	<0.001
	MB	25 (36.2)	10 (21.3)	15 (68.2)	
	Other	35 (50.7)	34 (72.3)	1 (4.5)	
WHO grade, *n* (%)	Low	34 (49.3)	17 (36.2)	17 (77.3)	0.003
	High	35 (50.7)	30 (63.8)	5 (22.7)	
Oropharyngeal dysfunction, *n* (%)	No	53 (76.8)	45 (95.7)	8 (36.4)	<0.001
	Yes	16 (23.2)	2 (4.3)	14 (63.6)	
Behavior change, *n* (%)	No	36 (52.2)	33 (70.2)	3 (13.6)	<0.001
	Yes	33 (47.8)	14 (29.8)	19 (86.4)	
Radiotherapy	No	37 (53.6)	20 (42.6)	17 (77.3)	0.015
	Yes	32 (46.4)	27 (57.4)	5 (22.7)	
Chemotherapy	No	35 (50.7)	19 (40.4)	16 (72.7)	0.025
	Yes	34 (49.3)	28 (59.6)	6 (27.3)	
Time since surgery, mean (SD)		45.2 (16.7)	42.2 (17.5)	51.6 (12.7)	0.015

CMS, cerebellar mutism syndrome; VP shunt, ventriculoperitoneal shunt; EOR, extent of resection.

### Outcomes of QoL

The median follow-up period between surgery and this assessment was 2.5 years. Children diagnosed with CMS had significantly lower total and subdomain scores than non-CMS children (*p* < 0.05), except for emotional scores (*p* > 0.05) (Table 3).

**Table 3:** comparison of QOL between CMS and non-CMS patients.

	Overall (*N* = 69), mean (SD)	non-CMS (*N* = 47), mean (SD)	CMS (*N* = 22), mean (SD)	*p*-Value
Physical score	76.6 (22.2)	85.3 (16.8)	57.8 (20.8)	**<0.001**
Emotional score	81.9 (13.5)	83.7 (14.7)	78.0 (9.6)	0.062
Social score	80.1 (18.2)	85.1 (16.6)	69.5 (17.2)	**0.001**
Academic score	73.7 (20.3)	79.9 (17.1)	60.5 (20.7)	**0.001**
Total score	77.9 (16.8)	83.7 (14.8)	65.3 (14.2)	**<0.001**

CMS, cerebellar mutism syndrome. The bold values significant in the statistic test.

The items in each domain were compared between the two groups (Table 4). Among the 23 items, children with CMS scored significantly lower on 15 items than the non-CMS children. In the emotional domain, score of Item E2 was significantly lower in children with CMS than non-CMS children (3.2 ± 0.8 vs. 3.7 ± 0.9, *p* = 0.023).

**Table 4:** Items comparison between CMS and non-CMS patients.

	Overall (*N* = 69)	non-CMS (*N* = 47)	CMS (*N* = 22)	*p*-Value
P1, mean (SD)	82.3 (25.8)	89.8 (18.6)	66.4 (31.7)	**0.003**
P2, mean (SD)	72.5 (30.9)	83.4 (23.3)	49.1 (32.5)	**<0.001**
P3, mean (SD)	68.4 (31.5)	77.9 (28.0)	48.2 (29.4)	**<0.001**
P4, mean (SD)	74.8 (26.5)	83.4 (21.8)	56.4 (26.6)	**<0.001**
P5, mean (SD)	81.2 (25.9)	91.1 (17.1)	60.0 (28.9)	**<0.001**
P6, mean (SD)	77.4 (27.2)	88.9 (17.6)	52.7 (28.0)	**<0.001**
P7, mean (SD)	80.9 (17.6)	84.3 (18.1)	73.6 (14.3)	**0.011**
P8, mean (SD)	75.1 (24.6)	83.8 (19.8)	56.4 (23.6)	**<0.001**
E1, mean (SD)	80.6 (18.5)	81.3 (19.3)	79.1 (16.9)	0.635
E2, mean (SD)	81.4 (18.6)	84.3 (19.1)	75.5 (16.3)	0.053
E3, mean (SD)	71.3 (18.3)	74.5 (19.0)	64.5 (15.0)	**0.023**
E4, mean (SD)	88.7 (14.7)	90.2 (15.5)	85.5 (12.6)	0.182
E5, mean (SD)	87.2 (15.7)	88.1 (17.0)	85.5 (12.6)	0.476
S1, mean (SD)	82.3 (19.9)	85.1 (20.6)	76.4 (17.1)	0.070
S2, mean (SD)	80.9 (20.4)	84.3 (19.1)	73.6 (21.7)	0.057
S3, mean (SD)	89.0 (14.8)	90.2 (15.5)	86.4 (12.9)	0.286
S4, mean (SD)	76.8 (25.6)	85.1 (20.6)	59.1 (26.5)	**<0.001**
S5, mean (SD)	71.6 (26.4)	80.9 (21.7)	51.8 (25.2)	**<0.001**
A1, mean (SD)	74.8 (23.7)	79.1 (22.8)	65.5 (23.2)	**0.027**
A2, mean (SD)	72.8 (21.7)	75.3 (21.4)	67.3 (21.9)	0.159
A3, mean (SD)	74.5 (24.7)	82.1 (22.9)	58.2 (20.4)	**<0.001**
A4, mean (SD)	75.1 (27.1)	83.0 (20.0)	58.2 (32.6)	**0.003**
A5, mean (SD)	71.6 (27.5)	80.0 (21.3)	53.6 (31.1)	**0.001**

P, physical domain; E, emotional domain; S, social domain; A, academic domain. Statistical significance measures are highlighted in bold.

### Association between QoL and clinical variables

In the univariate analysis, the correlation or association between QoL score and clinical variables was presented with Pearson’s coefficient or difference of the mean values and *p* values. A weak negative correlation (−0.26 to −0.38, *p* < 0.05) was observed between age at surgery and QoL total score or its subdomain scores. Diagnosis of CMS, male sex, VP shunt placement, higher WHO tumor grade, radiotherapy, chemotherapy, tumor rest in midline location, and presence with hydrocephalus are associated with lower total score of QoL or sub-score of QoL (range of difference: −6.4 to −26.3, *p* < 0.05). Tumor size, extend of resection, and time to surgery were not associated with scores of QoL or its subdomains (*p* > 0.05) (see Table 5).

**Table 5:** Association of QOL and clinical variables.

	Total score	Physical score	Emotional score	Social score	Academic score
CMS versus non-CMS	**−18.4 (<0.001)**	**−27.5 (<0.001)**	−7.5 (0.062)	**−15.6 (0.001)**	**−19.4 (0.001)**
Age at surgery	**−0.28 (0.018)**	−0.19 (0.126)	**−0.26 (0.026)**	−0.23 (0.063)	**−0.38 (0.001)**
Male versus female	**−14.3 (<0.001)**	**−18.7 (<0.001)**	**−6.4 (0.042)**	**−15.4 (<0.001)**	**−14.2 (0.001)**
WHO grade (high vs. low)	**−16.4 (<0.001)**	**−23.0 (<0.001)**	**−8.2 (0.011)**	**−13.0 (0.002)**	**−17.2 (<0.001)**
Tumor size	−0.07 (0.556)	−0.14 (0.245)	−0.11 (0.377)	0.010 (0.938)	0.04 (0.767)
Tumor location (midline vs. lateral)	**−11.1 (0.011)**	**−16.4 (0.002)**	−3.8 (0.257)	−8.8 (0.122)	**−12.1 (0.022)**
Hydrocephalus (Yes vs. No)	−7.3 (0.067)	**−10.0 (0.050)**	−2.9 (0.420)	−7.0 (0.108)	−7.9 (0.089)
Extent of resection (GTR vs. non-GTR)	9.7 (0.121)	12.6 (0.119)	0.8 (0.846)	6.7 (0.308)	17.0 (0.052)
VP shunt placement (Yes vs. No)	**−17.7 (0.001)**	**−26.3 (0.001)**	**−6.5 (0.078)**	**−16.8 (0.001)**	**−15.8 (0.033)**
Radiotherapy (Yes/No)	**−17.9 (<0.001)**	**−25.2 (<0.001)**	**−9.5 (0.003)**	**−14.7 (0.001)**	**−17.7 (<0.001)**
Chemotherapy (Yes/No)	**−17.9 (<0.001)**	**−24.0 (<0.001)**	**−9.7 (0.003)**	**−14.8 (0.001)**	**−19.4 (<0.001)**
Time to surgery (month)	0.18 (0.132)	0.19 (0.115)	0.07 (0.547)	0.13 (0.280)	0.20 (0.104)

CMS, cerebellar mutism syndrome; GTR, gross total resection; VP shunt, ventriculoperitoneal shunt. The bold values significant in the statistic test.

Forward stepwise multivariable analyses were conducted to investigate independent risk factors of QoL total score and subdomain scores, and all variables in the univariate analysis were included. Diagnosis of CMS (coefficient: −8.61, 95%CI: [−16.95, −0.27], *p* = 0.043), older age at surgery (coefficient: −1.30, 95%CI: [−2.24, −0.36], *p* = 0.007), and receiving chemotherapy (coefficient: −9.82, 95%CI: [−17.48, −2.17], *p* = 0.013) were independently associated with lower total QoL score. CMS (coefficient: −15.31, 95%CI: [−25.77, −4.86]), VP shunt placement (coefficient: −12.86, 95%CI: [−24.07, −1.65], *p* = 0.025), and radiotherapy (coefficient: −13.63, 95%CI: [−23.32, −3.92], *p* = 0.007) were independently associated with lower physical scores. Chemotherapy (coefficient: −9.11, 95%CI: [−15.08, −3.14], *p* = 0.003) and age at surgery (coefficient: −0.96, 95%CI: [−1.85, −0.06], *p* = 0.037) were associated with emotional scores. Male sex (coefficient: −13.68, 95%CI: [−21.63, −5.75], *p* = 0.001) and VP shunt placement (coefficient: −14.36, 95%CI: [−24.20, −4.53], *p* = 0.005) were associated with lower social scores. Chemotherapy (coefficient: −17.45, 95%CI: [−25.28, −9.61], *p* < 0.001) and older age at surgery (coefficient: −1.92, 95%CI: [−3.10, −0.74], *p* = 0.002) were associated with lower academic scores, whereas extent of resection (coefficient: 12.16, 95%CI: [2.04, 24.29], *p* < 0.021) was associated with higher academic scores (Table 6). The multicollinearity diagnosis found no significant dependency among the predicted variables.

**Table 6:** Multivariable analysis for risk factors of PedsQL scores.

	Total score		Physical score		Emotional score		Social score		Academic score	
	Coeff (95% CI)	*p*	Coeff (95% CI)	*p*	Coeff (95% CI)	*p*	Coeff (95% CI)	*p*	Coeff (95% CI)	*p*
CMS versus non-CMS	−8.61 (−16.95 ~ −0.27)	0.043	−15.31 (−25.77 ~ −4.86)	0.005	—	—	—	—	—	—
Male versus female	—	—	—	—	—	—	−13.68 (−21.62 ~ −5.75)	0.001	—	—
Age at surgery	−1.30 (−2.24 ~ −0.36)	0.007	—	—	−0.96 (−1.85 ~ −0.06)	0.037	—	—	−1.92 (−3.10 ~ −0.74)	0.002
VP shunt placement	−10.14 (−18.87 ~ −1.40)	0.024	−12.86 (−24.07 ~ −1.65)	0.025	—	—	−14.36 (−24.20 ~ −4.53)	0.005	—	—
Chemotherapy	−9.82 (−17.48 ~ −2.17)	0.013	—	—	−9.11 (−15.08 ~ −3.14)	0.003	—	—	−17.45 (−25.28 ~ −9.61)	<0.001
Radiotherapy			−13.62 (−23.32 ~ −3.92)	0.007	—	—	—	—	—	—
Extent of resection (GTR/non-GTR)	—	—	—	—	—	—	—	—	13.16 (2.04 ~ 24.29)	0.021

CMS, cerebellar mutism syndrome; GTR, gross total resection; VP shunt, ventriculoperitoneal shunt.

## Discussion

CMS has been proposed to involve multiple domains, including speech, motor, emotion, and cognitive functions. Previous studies have concentrated on risk factors for CMS, whereas few studies have been conducted on children’s QoL. It has been suggested that patients with CMS should undergo routine follow-up to monitor emotional, behavioral, and social problems over time (Gora et al., 2017). In this cross-sectional study, we investigated the long-term QoL of children who underwent posterior fossa surgery and compared children with and without CMS. Our data showed that children with CMS had significantly lower QoL and subdomain scores than non-CMS children. No significant association was found between time to surgery and overall QoL scores. These findings stress the long-term negative influence of CMS, which is not only restricted to speech function.

Multivariable analysis was performed to eliminate the interference of other clinical variables, since patients with and without CMS were not evenly distributed with regard to clinical variables in the baseline comparison. This demonstrated that CMS was consistently associated with lower QoL and physical scores in univariable and multivariable analyses in our study. Children with CMS had the lowest score and greatest difference in physical function compared to non-CMS children. The observed weakness in physical function is consistent with previous findings (Hartley et al., 2019; Grieco et al., 2020), and should be given more attention.

The CMS group had significantly lower social score than the non-CMS group in the univariable analysis, despite this losing statistical significance in the multivariable analysis. This has been reported in survivors of other cancers; however, the CMS group seemed to face more difficulty in dealing with social relations (Barrera et al., 2005; Ness and Gurney, 2007; Lund et al., 2011). The cerebellum has been shown to participate in social functions (Cutando et al., 2022). Changes in dopamine D2 receptors levels in cerebellum of male mice during adulthood alter sociability and preference for social novelty (Cutando et al., 2022). Functional MRI studies have revealed three distinct representations for social function in cerebellum. Schmahmann holds that like coordinating the motor, the cerebellum modulates behavior, maintaining it around a homeostatic baseline appropriate to context (Guell et al., 2018). Previous studies based on semi-structured interviews found that survivors of CMS had difficulties maintaining social relations (Camara et al., 2020; Wibroe et al., 2021). In the multivariable analysis, male sex was independently correlated with lower social score. We will discuss this further below. Overall, our results suggest that more attention should be paid to psychological rehabilitation in children with CMS.

Cognitive impairment and physical dysfunction have significant impacts on school scores (Nesayan et al., 2019; Fritz et al., 2020). Processing speed is a cognitive ability reflecting the ability to automatically process information and to process information quickly and outside of conscious awareness. Previous studies have found that patients with CMS exhibit impaired (>2 SD below the mean value) processing speed and below-average intellectual ability at 1 year postoperatively, with scores remaining low over time (Schreiber et al., 2017). In our study, the CMS group had significantly lower school scores in the long-term assessment than the non-CMS group, which is consistent with previous studies, despite losing statistical significance in the multivariate analysis. However, Julia et al. (Grieco et al., 2020) reported that 18 pairs of patients with or without CMS showed no difference in overall intelligence, and expressive and receptive vocabulary (mean follow-up time, 3.26 years). Further studies on this field may provide more compacted evidence.

Persistent emotional and behavioral dysregulation is another concern in children with CMS (Steinbok et al., 2003; Lanier and Abrams, 2017; Schreiber et al., 2017). In our study, no significant difference in emotional scores was found between patients with and without CMS, which is consistent with previous studies (Grieco et al., 2020). However, the analysis of each item of the scale showed that children with CMS were more susceptible to loss of temper than children without CMS. This indicates that emotional dysregulation exists among children after posterior fossa surgery, but its relationship with CMS still needs to be investigated in further research.

Radiotherapy and chemotherapy were not consistently correlated with QoL scores (An et al., 2011; Kuhlthau et al., 2012; Bull et al., 2014). There are studies (Penn et al., 2009; Aukema et al., 2013) that found no significant differences in the QoL of children who received surgery only and those who received surgery with adjuvant therapy. In contrast, a series of studies (Pogorzala et al., 2010; Kuhlthau et al., 2012; Netson et al., 2016) demonstrated the detrimental effects of radiotherapy or chemotherapy on QoL. In our study, chemotherapy and radiotherapy were related to lower QoL score and subdomain scores in both univariate and multivariate analyses. The detrimental effects of chemotherapy or radiotherapy may persist long term (Prabhu et al., 2014; Lawrie et al., 2019).

Male participants had poorer QoL scores across all subdomains than female participants in the univariable analysis in our study. However, in the multivariable analysis, sex was exclusively significantly associated with social scores, rather than QoL total score and other sub-domains. The relationship between sex and QoL in pediatric patients with brain tumors remains controversial (Bell et al., 2018). Previous reports indicated that males had lower school-related QoL score (Bhat et al., 2005; Lipak et al., 2022; Ljungman et al., 2022). However, there were also reports that women had poorer QoL scores than men, or that no significant difference existed between males and females (Bull et al., 2014; Barrera et al., 2017; Quast et al., 2018). No evidence has shown the sex effect for social performance in children with brain tumor (Litzelman et al., 2011). Previous studies have indicated the increased prevalence of CMS in males in contrast to females (Robertson et al., 2006; Gora et al., 2017; Yang et al., 2022a). Considering the male’s predominance in the CMS group, it is reasonable to presume that the lower QoL score and sub-domain scores are associated with CMS. And given the small sample size of our data, the impact of sex on QoL still needs to be validated in future studies.

Age at diagnosis was negatively associated with QoL scores in our results, which means patients receiving surgery at an older age have more deteriorative QoL scores. This was inconsistent with previous studies (Mulhern et al., 1992; Silber et al., 1992; Mulrooney et al., 2019; Sleurs et al., 2022). It was perceived that excessive treatment, including radiotherapy and chemotherapy, had a greater negative effect when children were younger. Mulhern et al. (1992) and Silber et al. (1992) found that Younger age with irradiation treatment was associated with lower intelligent performance. However, a systematic review demonstrated that older age at diagnosis was related to decreased QoL scores (Macartney et al., 2014). Although the harmful effect of radiotherapy and chemotherapy at a younger age is inevitable, the restoration ability of the somatic body decreases with age. With regard to the total QoL score, considering the capacity of compensation of all physical domains decreases gradually with age, younger age is expected to be a favorable predictor of QoL.

Hydrocephalus has been associated with inferior neurocognitive outcomes (Di Pinto et al., 2012; Brinkman et al., 2016). VP shunts are thought to be associated with favorable long-term QoL, since it addresses the increased intracranial pressure (Reimers et al., 2009). However, for children with brain tumors, this association may not hold true. In our study, VP shunt placement is a predictor of lower QoL scores. In our center, the removal of tumors is the highest priority to address the increased cranial pressure, rather than the VP shunt. The various complications associated with VP shunt should never be overlooked, and VP shunt placement should mainly be for patients with recurrent hydrocephalus after tumor resection. Therefore, patients with VP shunt placement may reflect worse medical experiences. Likewise, gross total resection was related to higher academic score, since non-gross total resection always occur when the tumor was infiltrative and malignant, and total resection cannot be easily performed.

No treatment or rehabilitation procedures for CMS have been established at present (Ashida et al., 2021). Treatment and rehabilitation should focus on three domains: language function, behavioral and cognitive function, and motor function. Pharmacological intervention, cognitive rehabilitation, and physical rehabilitation are the three main interventions at present. Several cases reported described mediation therapy such as corticosteroids, fluoxetine, thyrotropin-releasing hormone, bromocriptine and zolpidem, and modafinil, which were used in the acute stage to alleviate the symptoms of CMS (Catsman-Berrevoets, 2017; Molinari et al., 2019; Paquier et al., 2020). However, the results were inconsistent and not proven by larger-scale trials. Besides, speech therapists might help address mutism symptoms. Earlier intervention may provide the opportunity for avoiding the eventual emergence of long-term sequelae (Paquier et al., 2020). As regards behavioral and cognitive deficits, neuropsychological evaluation and the follow-up of long-term treatment are imperative for patients with CMS (Malbari et al., 2022). Studies have found that emotional intervention contributes to understand and manage emotions, and this may relive the emotional irritability in CMS children (Ratliff et al., 2022). This should be given more attention in future. The 2018 posterior fossa society meeting mentioned the opportunity of using transcranial direct current stimulation (tDCS) and transcranial magnetic stimulation (TMS) in the rehabilitation of CMS, which may resolve the diaschisis of the cerebellum and cerebrum. However, there is no direct evidence of the treatment effect of tDCS or TMS in patients with CMS. Future studies should pay more attention to this promising treatment.

Our study has several limitations. First, our study lacked baseline QoL assessment before surgery. As a result, we could not confirm that QoL scores of the children with CMS were worse than those of non-CMS children at baseline. Second, clinical variables were not evenly distributed between the two groups, especially because there were only two females in the CMS group, which may affect the representation of our results. Thirdly, the lack of information regarding the socioeconomic backgrounds of these families is another limitation of our study. The impact of socioeconomic background on rehabilitation treatment is important for the improvement of QoL score. In this study, we did not discuss the potential discriminative demographic and clinical variables between the groups, as only a proportion of the children with posterior fossa tumors in our center participated in the project, and the statistical analysis may contain sample biases. The small sample size may also result in selection bias. As for the risk factors of CMS, please refer to our previous studies, which enrolled a larger cohort (Yang et al., 2022a, b).

In summary, children with CMS exhibit poorer performance on QoL assessment, and motor dysfunction showed worse outcomes, which should be given more attention. The impact of the CMS on social and school performances cannot be ignored. Long-term neuropsychological assessment with individualized rehabilitation treatment is necessary to help these children return to a normal life. Although no significant difference was detected in the emotional scores, a significant difference in the outbreak of tempers was identified between the CMS and non-CMS groups. A more comprehensive investigation is needed to evaluate the emotional profile of patients after posterior fossa surgery.

## Data availability statement

The raw data supporting the conclusions of this article will be made available by the authors, without undue reservation.

## Ethics statement

The studies involving human participants were reviewed and approved by the Ethics Committee of Beijing Children’s Hospital. Written informed consent from the participants’ legal guardian/next of kin was not required to participate in this study in accordance with the national legislation and the institutional requirements.

## Author contributions

KZ, WY, ZY, YC, XP, NZ, HS, YJ, and MG contributed to the study conception and design. HS and YJ performed the material preparation. NZ, YC, and XP performed the data collection. ZY and WY performed the data analysis. WY and KZ wrote the first draft of the manuscript. MG conducted the funding acquisition and supervision. KZ, WY, ZY, YC, XP, NZ, HS, YJ, and MG commented on previous versions of the manuscript. All authors contributed to the article and approved the submitted version.

## Funding

This study was funded by Beijing Hospital’s Authority Clinical Medicine Development of Special Funding (code: XMLX202144) and Newborn Young Doctors Scientific Research Fund (Z-2019-41-2101-04).

## Conflict of interest

The authors declare that the research was conducted in the absence of any commercial or financial relationships that could be construed as a potential conflict of interest.

## Publisher’s note

All claims expressed in this article are solely those of the authors and do not necessarily represent those of their affiliated organizations, or those of the publisher, the editors and the reviewers. Any product that may be evaluated in this article, or claim that may be made by its manufacturer, is not guaranteed or endorsed by the publisher.

## Footnotes

^1^ https://www.posteriorfossasociety.org/

## Abbreviations

QoL: Quality of life
CMS: Cerebellar mutism syndrome
VP shunt: Ventroperitoneal shunt
PedsQL: Pediatric Quality of Life Inventory
WHO: World Health Organization
