# Supplementary material for: Long-term postoperative quality of life in childhood survivors with cerebellar mutism syndrome
Source: Front Psychol. 2023 Feb 24;14:1130331. doi: 10.3389/fpsyg.2023.1130331 (PMC9998537; doi:10.3389/fpsyg.2023.1130331)
Supplement: Supplementary file 1 [file Table_1.docx]

**Supplementary Table 1**

*In* *the* *past* ***ONE*** ***month,*** *how* *much* *of* *a* ***problem*** *has* *your* *child* *had* *with* *…*

| **PHYSICAL** **FUNCTIONING** ***(problems*** ***with…)*** | **Never** | **Almost** **Never** | **Some-**  **times** | **Often** | **Almost** **Always** |
| --- | --- | --- | --- | --- | --- |
| 1. Walking more than one block | 0 | 1 | 2 | 3 | 4 |
| 2. Running | 0 | 1 | 2 | 3 | 4 |
| 3. Participating in sports activity or exercise | 0 | 1 | 2 | 3 | 4 |
| 4. Lifting something heavy | 0 | 1 | 2 | 3 | 4 |
| 5. Taking a bath or shower by him or herself | 0 | 1 | 2 | 3 | 4 |
| 6. Doing chores around the house | 0 | 1 | 2 | 3 | 4 |
| 7. Having hurts or aches | 0 | 1 | 2 | 3 | 4 |
| 8. Low energy level | 0 | 1 | 2 | 3 | 4 |

| **EMOTIONAL** **FUNCTIONING** ***(problems*** ***with…)*** | **Never** | **Almost** **Never** | **Some-**  **times** | **Often** | **Almost** **Always** |
| --- | --- | --- | --- | --- | --- |
| 1. Feeling afraid or scared | 0 | 1 | 2 | 3 | 4 |
| 2. Feeling sad or blue | 0 | 1 | 2 | 3 | 4 |
| 3. Feeling angry | 0 | 1 | 2 | 3 | 4 |
| 4. Trouble sleeping | 0 | 1 | 2 | 3 | 4 |
| 5. Worrying about what will happen to him or her | 0 | 1 | 2 | 3 | 4 |

| **SOCIAL** **FUNCTIONING** ***(problems*** ***with…)*** | **Never** | **Almost** **Never** | **Some-**  **times** | **Often** | **Almost** **Always** |
| --- | --- | --- | --- | --- | --- |
| 1. Getting along with other children | 0 | 1 | 2 | 3 | 4 |
| 2. Other kids not wanting to be his or her friend | 0 | 1 | 2 | 3 | 4 |
| 3. Getting teased by other children | 0 | 1 | 2 | 3 | 4 |
| 4. Not able to do things that other children his or her age can do | 0 | 1 | 2 | 3 | 4 |
| 5. Keeping up when playing with other children | 0 | 1 | 2 | 3 | 4 |

| **SCHOOL** **FUNCTIONING** ***(problems*** ***with…)*** | **Never** | **Almost** **Never** | **Some-**  **times** | **Often** | **Almost** **Always** |
| --- | --- | --- | --- | --- | --- |
| 1. Paying attention in class | 0 | 1 | 2 | 3 | 4 |
| 2. Forgetting things | 0 | 1 | 2 | 3 | 4 |
| 3. Keeping up with schoolwork | 0 | 1 | 2 | 3 | 4 |
| 4. Missing school because of not feeling well | 0 | 1 | 2 | 3 | 4 |
| 5. Missing school to go to the doctor or hospital | 0 | 1 | 2 | 3 | 4 |
